# Relationship Between the Built Environment and Body Mass Index in a Rural Context: A Cross-Sectional Study from Vermont

**DOI:** 10.7759/cureus.3040

**Published:** 2018-07-24

**Authors:** Austin R Troy, Levi N Bonnell, Benjamin Littenberg

**Affiliations:** 1 Department of Planning and Design, University of Colorado, Denver, USA; 2 Department of Ophthalmology, University of Colorado School of Medicine, Aurora, USA; 3 General Internal Medicine Research, University of Vermont, Burlington, USA

**Keywords:** health geography, gis, built environment, rural health, building density, obesity, environmental epidemiology

## Abstract

Objective

To evaluate the association between a marker of urban development (commercial building density) and body mass index (BMI) in a predominantly rural context.

Methods

A cross-sectional analysis of two geocoded datasets from Vermont. The first includes subjects from the Vermont Diabetes Information System (VDIS), an extensively attributed dataset of adult diabetics (*n* = 610); the second was the complete driver's license records for Vermont (*n* = 401,367). The dependent variable was BMI, measured objectively for the VDIS data and self-reported for the driver's license data. The explanatory variable was commercial buildings per hectare within 250 m of the home address used as a proxy for walkability. We regressed BMI against density in both datasets, controlling for age and gender; a separate regression was run for the VDIS data, controlling for a number of additional confounders related to health, activity, diet, and income.

Results

All models demonstrated a significant positive relationship between BMI and commercial building density. For the three VDIS data models, coefficients of density were +0.75, +0.79, and +0.90, all of which indicate an approximate ¾ kg/m^2^ increase in BMI for each additional commercial facility per hectare (p < 0.01). For the driver’s license data, the coefficient was +0.16, which also indicates an increase in BMI with increasing density (p < 0.01).

Discussion

We found that BMI displays a positive association with commercial building density in Vermont, which is inconsistent with previous findings. The difference may be due to the unique rural focus of this study. Other characteristics of rural life may be associated with lower incidence of obesity and should be studied further.

## Introduction

Obesity, defined as a body mass index (BMI) of greater than or equal to 30 kg/m^2^, is a significant risk factor for morbidity and mortality [1]. An additional 5 kg/m^2^ increase in BMI is associated with up to 30% higher mortality [2]. The development of obesity is not fully understood but can be partially explained by environmental factors that promote or impede physical activity and access to healthy food [3-4].

Research suggests that greater walkability results in decreased BMI in metropolitan and urban settings. Walkability is conceptualized as having three components: density, diversity of land use, and design [5-6]. Residential density, degree of land use mix, and street connectivity were associated with increased walking and decreased incidence of overweight and obesity among men in metropolitan Atlanta [7]. Population density and land use mix were both negatively associated with BMI in New York City [8]. Land use mix and street connectivity were associated with a decrease in overweight/obesity and an increase in walking activity in Portland, Oregon [9]. A composite index of walkability (based on average block size, frequency of small blocks, and number of intersections) correlated positively with the amount of walking in major metropolitan counties in the United States, but there was only a weak relationship with BMI [10]. Finally, the presence of transit stops, an indicator of lower automobile dependency, is associated with lower BMI, [8-9, 11]. Similar results have been found in other countries [12-14].

The literature, however, is far from being in total agreement. Lovasi et al. found that walking was predicted by neither land use diversity nor density in western Washington state [11], while Smith et al. found only a limited relationship between population density and body weight in Salt Lake County [6]. When stratified by socio-economic status, land use mix and population density were associated with lower BMI among those with higher education and income, but not among socio-economically disadvantaged groups in New York City [15].

One contextual factor that has received little attention in this literature is the rural-urban spectrum. A review of the literature on built environment and BMI included 68 papers, of which 60 studied metropolitan areas, seven studied rural areas, one studied an exurban area, and none studied small towns [16]. This disparity is striking because rural adults tend to be more obese than their urban counterparts in the United States. A recent analysis found that 39.6% of rural adults were obese compared to 33.4% of urban adults in the United States [17]. This relationship between rurality and obesity is not necessarily true in other countries [18]. Among the few rural studies completed in the United States, Boehmer et al. found both distance to recreational facilities and fear of safety predict BMI independent of dietary fat, sedentary behavior and physical activity in rural Missouri, Tennessee, and Arkansas [19].   

Although evidence suggests that the built environment has an impact on BMI, the relationship is poorly understood in rural areas. We sought to better understand the relationship between the built environment and obesity in a nonmetropolitan region by investigating the relationship between BMI and commercial building density in Vermont.

## Materials and methods

Study area

The study area was the state of Vermont. With 630,000 inhabitants, it has the second smallest population of any state, and ranks 30th in the nation for overall population density. Its largest city, Burlington, has approximately 38,000 residents, and is in the state’s only metropolitan area. With fewer than 150,000 residents, the metro area is smaller and less urban than those described in much of the literature. The 2010 US Census reports that Vermont’s population was 34% urban and 66% rural, defined as not an urbanized area (50,000 people or more) or an urban cluster (greater than 2,500 and less than 50,000 people).

Sample

Two datasets were used. The first was from the Vermont Diabetes Information System (VDIS). VDIS is a regional registry and quality improvement system for primary care providers and their adult patients with diabetes. The registry includes 64 selected nonacademic primary care practices and 7,414 patients located in all areas of the region. A random sample of study patients were invited to participate in a field survey involving a home visit [20]. Seven hundred and twenty-four Vermont adults with diabetes were included.

Each subject completed a questionnaire concerning their medical history, use of medications, physical activity, and other personal characteristics that could possibly confound the relationship between commercial building density and body mass including age and gender. Household income was collected in seven categories and classified as low (below the median of $30,000 per year) versus high. The number of medications used per day was taken as a marker of treatment burden. Alcohol use was considered present if the subject endorsed “Do you currently drink alcohol?” and smoking if they endorsed “Have you smoked a cigarette – even one puff – during the past seven days?” Physical exercise and diet were recorded as the self-reported percentage of days in the last week that the subject followed recommendations for adults with diabetes. Trained field assistants measured weight using a portable spring-loaded scale (LB Dial Scale HAP200-41, Healthometer, Inc, McCook, IL) and height using a portable stadiometer (SECA, Inc, Chino, CA). Blood pressure was measured three times with an automatic sphygmomanometer (Omron model #HEM-711, Omron Healthcare Inc, Vernon Hills, IL). Diabetes control was measured by obtaining the most recent report of hemoglobin A1C from the patient’s clinical laboratory. A1C is proportional to the average blood sugar over the previous six weeks, with higher numbers indicating poorer control.

The second dataset included driver’s license records (and nondriving identity cards) for Vermont residents, obtained with permission from the Department of Motor Vehicles [21]. These data included over 577,000 records of self-reported height and weight, age, gender, and home address collected between the years 2000 and 2014. Self-reported height and weight data may be updated at the time of license renewal, which varies state-to-state.

Analysis

We used the ArcGIS Address Coder (GIS Software, Environmental Systems Research Institute, Inc., Redlands, CA) to geocode participants’ home addresses to longitude and latitude coordinates. Ninety-six VDIS observations were not successfully geocoded and 17 were discarded due to nonresponses for needed data fields, leaving 611 analyzable subjects. Fifty-two of these did not report their income and were excluded from one of the models. For the driver’s license data, geocoding yielded 401,367 records for a success rate above 70%.

We estimated commercial building density at four different neighborhood sizes (i.e., spatial scales) defined by circles with radii of 250, 500, 1000, and 2000 m, with each circle representing a different spatial denominator over which density was calculated. We experimented with four different neighborhood sizes because there is no clear guidance in the literature on the spatial scale for defining walkability. We used the ArcGIS Point Density function, which calculates density for each pixel by creating a circular neighborhood with user defined radius centered on that pixel, counting the number of points within that circle and dividing by area. This process is done for every pixel on the map, resulting in a continuous density surface with a resolution of 30 m. Density values from the resulting map were then spatially assigned to the geocoded address points using the zonal statistic function. Building data needed for the density calculation were obtained from the 2006 Vermont e911 emergency response database (Vermont Center for Geographic information, Montpellier, VT). We chose commercial building density other than a composite score of walkability because we theorized it would be a valid measure of destinations, a key component of walkability [22].

Each subject’s BMI was regressed, using ordinary least squares, against the building density associated with their home address, while controlling for several potential confounders. In the case of the driver’s license data, the only potential confounders available were age and gender, as given in model D1. For comparative purposes, we ran a similar model for VDIS, including just density, age, and gender as independent variables (model V1). We included a quadratic term for age in both regressions, based on previous research showing a concave-down relationship between prevalence of obesity and age, where BMI peaks in late middle age [23].

We also ran two more extensive models using the VDIS data, accounting for a larger number of potential confounders including exercise, diet, age, sex, race, smoking, drinking, diabetic control by A1C, blood pressure, and number of daily medications, all of which were expected to influence BMI (model V2). We repeated the analysis with the smaller sample that had income data (model V3). Many transformations of the dependent variable were attempted, but regression diagnostics indicated that these did not significantly benefit the model in terms of fit or homoscedasticity. Variance inflation factors were all less than 2.0, suggesting multi-collinearity was not problematic.

For the VDIS models, we also ran a spatial error regression with a neighborhood matrix based on the three nearest neighbors to test the robustness of results to spatial autocorrelation [24].  Strong spatial autocorrelation, particularly in the error term, may represent a form of pseudo-replication, increasing the chance for type 1 error. This model helps determine if the null hypothesis has been falsely rejected due to spatial autocorrelation in regression models and is a more conservative form of hypothesis testing. Using a number of different neighbor weight matrices, we tested whether adjustment for autocorrelation was necessary and found that it was not for models V2 and V3, based on Lagrange Multiplier and Robust Lagrange Multiplier tests of significance. Model V1, however, did yield a Robust Lagrange Multiplier test statistic of 6.6 (p = 0.01) for the spatial error model, so we ran a spatial error regression on V1, using several neighbor thresholds, from 250 to 500 m [25]. In no case did the sign or significance of any variables change. Therefore, we present only the linear model results. Spatially adjusted regression is not presented for model D1 because of computational constraints in processing such a large dataset.

## Results

Subjects from the driver’s license data set had lower BMI, lived in less commercially dense areas, were younger and more often female than the diabetic subjects (Table 1).

**Table 1:** Characteristics of subjects included in the final models. Model D1 uses the driver’s license data; models V1-V3 use the Vermont Diabetes Information System. V3 is a subset of V1 with income data. *σ*: standard deviation; A1C: hemoglobin A1C.

		Model D1			Models V1 and V2		Model V3	
		*n* = 401,367			*n* = 611		*n* = 559	
Variable	Definition	Mean	*σ*	Mean		*σ*	Mean	*σ*
Body mass index	Weight in kilograms divided by height in meter squared	25.1	4.5	33.4		6.9	33.4	6.9
Density	Number of commercial buildings within 250 m	0.18	0.56	0.30		0.74	0.32	0.76
Age	Age in years	42.6	17.4	65.1		12.1	64.7	12.1
Sex	Male = 1; Female = 0	0.33	-	0.52		-	0.52	-
Drinker	Drink alcohol currently = 1; all others = 0	-	-	0.28		-	0.27	-
Blood pressure	Diastolic blood pressure in mmHg	-	-	78.4		10.8	78.4	10.9
A1C	Blood glycosylated hemoglobin A1C (%)	-	-	7.09		1.35	7.11	1.36
Diet	Self-reported percentage of days in the last week that the subject followed their recommended diet plan	-	-	57.4		-	57.6	-
Exercise	Self-reported percentage of days in the last week that the subject performed the recommended amount of exercise	-	-	34.5		-	33.1	-
Smoker	Smoke cigarettes currently = 1; all others = 0	-	-	0.16		-	0.16	-
Medication count	Number of medications used daily	-	-	8.7		4.5	8.7	4.5
Low income	Income < $15,000/year = 1; all others = 0	-	-	-		-	0.31	-

Regression results of all three models are provided in Table 2. Using a backwards stepwise approach, variables not significant at the 90% confidence level for at least one of the models were removed. Of the building densities mentioned above, only commercial density at the 250-m search radius scale was significant in both datasets, so only it is included in the models presented below.

**Table 2:** Multivariate least-squares regression on body mass index. Model D1 uses the driver’s license data; models V1-V3 use the Vermont Diabetes Information System. *β*: beta-coefficient from multiple linear regression controlling for all the variables listed; A1C: hemoglobin A1C.

	Model D1		Model V1		Model V2		Model V3	
Variable	*β*	p	*β*	p	*β*	p	*β*	p
Commercial density (ha^-1^)	0.16	<0.001	0.75	0.03	0.90	0.007	0.79	0.02
Age (years)	0.21	<0.001	0.71	<0.001	0.64	<0.001	0.69	<0.001
Agesquared (years^2^)	-0.002	<0.001	-0.007	<0.001	-0.006	<0.001	-0.007	<0.001
Male sex	2.13	<0.001	2.76	<0.001	1.89	0.001	1.82	0.001
Drinker					-1.56	0.006	-1.36	0.002
Blood pressure (mmHg)					0.11	<0.001	0.12	<0.001
A1C (%)					0.56	0.003	0.59	0.002
Diet (% of days)					-0.02	0.03	-0.02	0.02
Exercise (% of days)					-0.03	<0.001	-0.03	<0.001
Smoker					-1.86	0.007	-1.94	0.007
Medication count					0.21	<0.001	0.19	0.002
Low income							1.65	0.005
*R*^2^	0.12		0.16		0.26		0.27	
*n*	401,367		611		611		559	

Across models, most variables had the expected sign (Table 2): lower BMI was associated with lower blood pressure, better diabetic control, better adherence to dietary and exercise guidelines, smoking, fewer medications, and higher income. The vast majority of drinkers in the VDIS sample were light or moderate drinkers, which has been previously described to be associated with lower BMI [26]. However, female gender was associated with lower BMI, which is inconsistent with other findings [3].

As indicated by the statistically significant squared term, age was quadratically related to BMI, in an inverse-U shape, consistent with the literature discussed above [23]. In model D1, predicted BMI peaks at 64 years, while in models V1-V3, it peaks between ages 51 and 53.

The coefficient on commercial density ranged from 0.157 (D1) to 0.898 (V2) and was significant at the 95% confidence level for all models, meaning that when controlling for potential confounders, BMI was significantly lower among subjects living in neighborhoods with lower commercial building density.

Spatial error regression models for VDIS data obtained nearly the same results. All variables retained the same significance and sign with only slight differences in magnitude. This finding suggests that the VDIS models are robust to spatial effects.

## Discussion

In these data, higher commercial density was associated with higher BMI, holding all else constant. Because density was measured as the number of commercial facilities per hectare (for the 250-m area around the patient’s address), the coefficient of 0.78 in model V3 can be interpreted as about a 3/4 kg/m^2^ increase in BMI for each additional commercial facility per hectare. This is a large number (over 5-1/2 pounds for a six-foot adult) with substantial clinical and public health implications. The coefficient is smaller for the driver’s license data (0.16). This may be due to the lack of controlling for confounders in the D1 model, self-reporting measurement error, differences in the mean commercial density, other unmeasured contextual factors between the two groups, or that VDIS represents an older, chronically ill portion of the population. In either case, both results can be interpreted as indicating at least a one-pound increase associated with each additional commercial building per hectare.

Much of the literature has found either that urban sprawl is positively associated with BMI or that compactness and density are associated with lower BMI [7]. However, other studies show that the density-obesity relationship can be mediated by context [6, 14]. In those two studies, that contextual difference is socio-economic. In our study, it appears to be the urban-rural gradient. These results suggest that the characteristics of the built environment that correlate with physical activity may be different outside of major metropolitan areas with large urban cores, which is the setting for most of the previous literature. 

**Figure 1:**
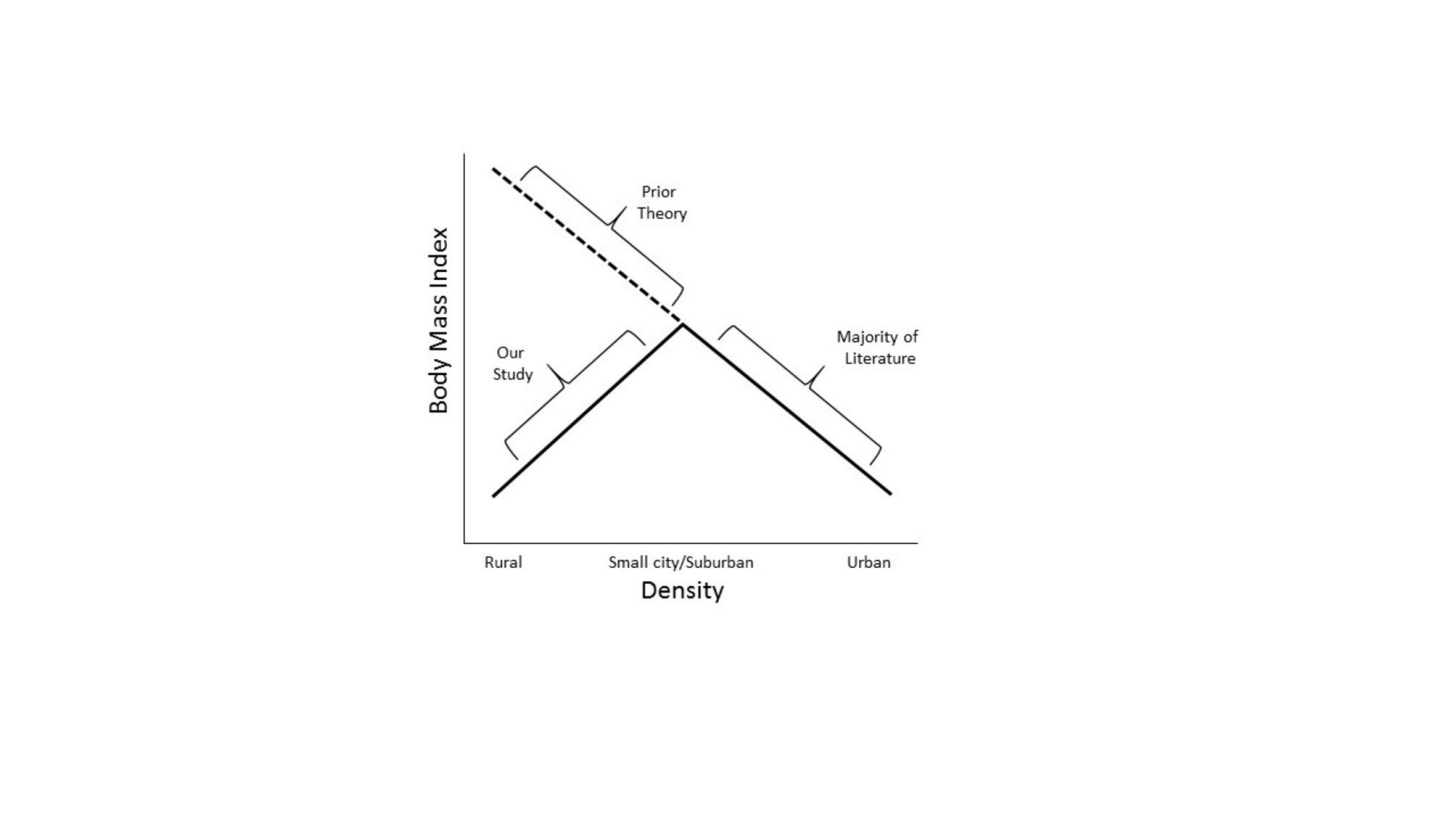
Expected effects of density of the rural–urban spectrum on obesity.

The relationship between BMI and density may be nonlinear over a large spectrum of urbanization, with a positive relationship in low density areas and a negative relationship in urban areas (Figure 1). Few studies have explored the left side of the graph, and none has adequately looked at the full urbanization gradient at once.

There are several possible explanations for why a positive association between commercial density and BMI is found in Vermont while the opposite is found in many large metropolitan areas (at least among higher SES groups). In metropolitan areas with big cities, the BMI-density relationship largely has to do with active transport. Significant portions of the populace walk on a regular basis as part of commuting and shopping, either because walking is made easy through proximity of attractive destinations and pedestrian design, because driving is expensive and hard, or because of the wider availability of mass transit, which typically involves at least some walking to connect trip segments. Marginal increases in density in such a context can then result in perceptible decreases in automobile dependency and more physical activity. Therefore, density, or similar urban measures, can act as a proxy for walkability.

In a less urbanized setting like Vermont, the settlement pattern is so dispersed and dependence on automobiles so overwhelming that marginal differences in density are unlikely to be associated with significant differences in active transport.

This explains why density would not be negatively associated with BMI, but why would there be a positive association?  Perhaps, just as high commercial density is a proxy for walking in urban areas, low commercial density acts as a proxy measure for physical activities more common in rural areas. While some previous research has found that rural residents tend to be heavier than comparable urban populations, research has also found tremendous variation in the determinants of BMI and physical activity amongst rural subjects across different regions [27].

Our results suggest that rural Vermonters are more active than those living in more suburban contexts. Vermont’s rural residents have excellent access to open space and outdoor recreational facilities, such as hiking trails, ski areas, paddling routes, lake shores, etc. Further, rural occupations, such as farming and logging, tend to be physically active.  The low incidence of obesity in rural Amish farming families appears due to extremely high levels of farm-related physical activity [28]. Rural property owners also tend to have much larger lots, requiring more physically intensive property maintenance activities, such as wood splitting, brush clearance, gardening, snow removal, and mowing than in other areas. Under this explanation, low density serves as a marker of increased nonwalking physical activity, while in bigger metropolitan areas, density acts more as a measure of walkability.

These analyses have several important limitations. The VDIS included only adults with diabetes, a condition associated with obesity and reduced physical activity. The driver’s license data are more representative of the general population (virtually every adult in the state, including undocumented immigrants, is eligible for a license or identification card), but the data are subject to self-report bias. Presumably, many adults overestimate their height and underestimate their weight. Although this biases the reported BMI, it will not confound the relationship between BMI and density unless the degree of bias is related to density. We believe it very unlikely that residents living close to commercial buildings are more or less likely to misreport their weight than those living in less building-dense areas. It is reassuring that the coefficients on commercial density are of the same sign and significance in all models, although they vary in magnitude, suggesting that these analyses are robust in spite of these limitations, and that driver’s license data are useful in this analytic role. Both datasets were drawn from Vermont, a state that may not be representative of other rural areas across the country. Geocoding errors tend to be larger in rural areas compared to suburban and urban areas [29].

Strengths of this study include a well-characterized sample of subjects with objectively measured BMI, and a broadly generalizable population in the driver’s license data. The consistency of the various analyses suggests robust findings. Finally, this is one of the first studies to look at compactness of the built environment and obesity in a predominantly rural area.

## Conclusions

Contrary to previous findings in predominantly urban settings, we found that compactness of the built environment was associated with an increase in BMI in a largely rural state. Differences in context (rural versus urban settings) influence the relationship of individual aspects of the built environment (such as commercial building density) with human health (such as obesity) such that solutions that may work in suburban and urban areas (such as increasing commercial density or other incentives to active transport) may actually be counterproductive in rural areas.

## References

[1] Eknoyan G (2008). Adolphe Quetelet (1796-1874) - the average man and indices of obesity. Nephrol Dial Transplant.

[2] Whitlock G, Lewington S, Sherliker P (2009). Body-mass index and cause-specific mortality in 900 000 adults: collaborative analyses of 57 prospective studies. Lancet.

[3] Rodgers A, Woodward A, Swimburn B, Dietz W (2018). Prevalence trends tell us what did not precipitate the US obesity epidemic. Lancet Pub Health.

[4] Swimburn BA, Sacks G, Hall KD (2011). The global obesity pandemic: shaped by global drivers and local environments. Lancet.

[5] Cervero R, Kockelman K (1997). Travel demand and the 3Ds: density, diversity, and design. Transport Res Part D - Travel Environ.

[6] Smith KR, Brown BB, Yamada I, Kowaleski-Jones L, Zick CD, Fan JX (2008). Walkability and body mass index density, design, and new diversity measures. Am J Prev Med.

[7] Frank LD, Kerr J, Sallis JF, Miles R, Chapman J (2008). A hierarchy of sociodemographic and environmental correlates of walking and obesity. Prev Med.

[8] Rundle A, Diez Roux AV, Free LM, Miller D, Neckerman KM, Weiss CC (2007). The urban built environment and obesity in New York City: a multilevel analysis. Am J Health Promot.

[9] Li F, Harmer PA, Cardinal BJ (2008). Built environment adiposity, and physical activity in adults aged 50-75. Am J Prev Med.

[10] Doyle S, Kelly-Schwartz A, Schlossberg M, Stockard J (2006). Active community environments and health: the relationship of walkable and safe communities to individual health. J Am Plann Assoc.

[11] Lovasi GS, Moudon AV, Pearson AL (2008). Using built environment characteristics to predict walking for exercise. Int J Health Geogr.

[12] Morency C, Trépanier M, Demers M (2011). Walking to transit: an unexpected source of physical activity. Transp Policy.

[13] Dempsey S, Lyon S, Nolan A (2018). Urban green space and obesity in older adults: evidence from Ireland. SSM Popul Health.

[14] Oliver M, Witten K (2015). Neighbourhood built environment associations with body siz in adults: mediating effects of acticity and sedentariness in a cross-sectional study of New Zealand adults. BMC Public Health.

[15] Lovasi GS, Neckerman KM, Quinn JW, Weiss CC, Rundle A (2009). Effect of individual or neighborhood disadvantage on the association between neighborhood walkability and body mass index. Am J Public Health.

[16] Feng J, Glass TA, Curriero FC, Stewart WF, Schwartz BS (2010). The built environment and obesity: a systematic review of the epidemiologic evidence. Health Place.

[17] Befort CA, Nazir N, Perri MG (2012). Prevalence of obesity among adults from rural and urban areas of the United States: findings from NHANES (2005‐2008). J Rural Health.

[18] Angkurawaranon C, Jiraporncharoen W, Chenthanakij B, Doyle P, Nitsch D (2014). Urban environments and obesity in Southeast Asia: a systematic review, meta-analysis and meta-regression. PLOS One.

[19] Boehmer TK, Lovegreen SV, Haire-Joshu D, Brownson RC (2006). What constitutes an obesogenic environment in rural communities?. Am J Health Promot.

[20] MacLean CD, Littenberg B, Gagnon M, Reardon M, Turner PD, Jordan C (2004). The Vermont Diabetes Information System (VDIS): study design and subject recruitment for a cluster randomized trial of a decision support system in a regional sample of primary care practices. Clin Trials.

[21] Littenberg B, Lubetkin D (2016). Availability, strengths and limitations of US state driver’s license data for obesity research. Cureus.

[22] Brown BB, Yamada I, Smith KR, Zick CD, Kowaleski-Jones L, Fan JX (2009). Mixed land use and walkability: variations in land use measures and relationships with BMI, overweight, and obesity. Health Place.

[23] Flegal KM, Carroll MD, Ogden CL, Johnson CL (2002). Prevalence and trends in obesity among US adults, 1999-2000. J Am Med Assoc.

[24] Cliff AD, Ord JK (1981). Spatial Processes: Models and Applications.

[25] Anselin L (1988). Lagrange multiplier test diagnostics for spatial dependence and spatial heterogeneity. Geogr Anal.

[26] Breslow RA, Smothers BA (2005). Drinking patterns and body mass index in never smokers: National Health Interview Survey, 1997-2001. Am J Epidemiol.

[27] Casey AA, Elliott M, Glanz K (2008). Impact of the food environment and physical activity environment on behaviors and weight status in rural U.S. communities. Prev Med.

[28] Basset DR, Schneider PL, Huntington GE (2004). Physical activity in an old order Amish community. Med Sci Sports Exerc.

[29] Cayo MR, Talbot TO (2003). Positional error in automated geocoding of residentaial addresses. Int J Health Geogr.

